# Stress distribution on platform switched dental implant technique with different bone type

**DOI:** 10.6026/973206300214539

**Published:** 2025-12-15

**Authors:** Inamuddin Inamuddin, Suman Yadav, Geeta Sharma, Randhir Kumar, Sharib Abdus Salam

**Affiliations:** 1 Department of Oral and Maxillofacial Surgery, Dr. R Ahmed Dental College and Hospital, Kolkata, West Bengal, India; 2 Department of Oral Surgery, ITS Dental College, Muradnagar, Ghaziabad, Uttar Pradesh, India; 3 Department of Oral Pathology, Patna Dental College & Hospital, Patna, Bihar, India; 4 Department of Periodontology, Patna Dental College & Hospital, Patna, Bihar, India; 5 Department of Periodontics, Patna Dental College and Hospital, Patna, Bihar, India

**Keywords:** Dental implant, flat form switching, stress

## Abstract

The concept of platform switching has been introduced to implant dentistry based on clinical observations for reduced peri-implant crestal bone loss. Therefore, it is of interest to assess the platform switching technique's impact on stress distribution across various bone types. Implants measuring 3.0 mm, 4.3 mm, and 5.0 mm in diameter and 11 mm in length were selected and positioned on both D2 and D3 bone models. Strain gauges were assessed with vertical load on the buccal and lingual sides of each sample. Data shows that bone strain values are high on the buccal side.

## Background:

It is common to place dental implants to replace one or more missing teeth [1, 2, 3-4]. Implant-supported prostheses have become a widely accepted option for replacing missing posterior teeth due to durability and ability to restore masticatory function [5]. The marginal bone level surrounding the implant is used to evaluate the success of the prosthesis. The Loss of marginal bone is a complex issue that involves multiple mechanical and biological factors [6]. Factors related to biomechanics that affect the stress experienced by bone surrounding an implant include; the characteristics and amount of bone, design and diameter of the implant, and platform switching [7]. Vertical and transverse loads from mastication act on implant-supported prostheses, resulting in axial forces and bending moments that concentrate stress on both the implant and the surrounding bone [8]. Load transfer from implants to the adjacent bone is affected by factors such as the loading type, bone-implant contact, implant dimensions, and bone quality and quantity [9]. Following implant surgery, changes in the bone level at the peri-implant crest take place [10]. Many factors influence the conservation of preimplant marginal bone loss. One of these is platform switching, which was introduced in the mid-1980s. Due to the fact that conventional implant abutment systems are typically flush with the implant shoulder, a micro crack can form between the implant and abutment when stress surpasses the yield strength, resulting in system failure. In platform switching, this issue is resolved by utilizing an abutment with a mismatch diameter. The concept of platform switching entails reducing the diameter of the restoration abutment in relation to that of the dental implant. Generally, an abutment of smaller diameter is used with an implant that provides a platform at the neck [11]. This setup leads to a circular horizontal step, which could allow for a horizontal expansion of the biological width. Platform switching is recommended to prevent or reduce crestal bone loss, in contrast to the conventional restorative procedure that uses an identical-sized implant and suprastructure diameter (standard platform) [12]. Platform switching (PS) has emerged as a promising biomechanical approach to maintain crestal bone levels, aiming to reduce marginal bone resorption and improve aesthetic results [13]. A systematic review and meta-analysis conducted by Atieh *et al.* concluded that, platform switching may help to preserve interimplant platform and reduce the extent of marginal bone resorption [14]. Therefore, it is of interest to assess the platform switching technique in relation to stress distribution across various bone types during dental implant placement.

## Materials and Methods:

In Prosthodontics department this *in vitro* study was conducted. The research was carried out on two distinct varieties of bone: D2 (which has greater density) and D3 (which has lesser density) simulators. This study utilized polyurethane blocks, which the American Society for Testing Materials (ASTM) has standardized for testing different densities and simulating D2 and D3 types of bones (trabecular bone). The density of the bones utilized in this research aligns with Misch's classification. Implants (Nobel Biocare, Göteborg, Sweden) with lengths of 11 mm and diameters of 3.0 mm, 4.3 mm, and 5.0 mm were used for the study. A solid polyurethane block with a density of 40 pcf (pound force per cubic foot) and dimensions of 2 x 2 x 6 cm was prepared for D2, and a synthetic cortical shell, 3 mm thick and commercially sourced, was affixed over it. In accordance with the manufacturer's specifications, a polyurethane block with a density of 10 pcf and identical dimensions was used to simulate the D3 bone. For each implant diameter, three specimens of artificial bones were prepared. Using the Paltop surgical kit (Paltop Master with drill stops kit), sequential osteotomy was performed on D2 and D3 bones, and implants with diameters of 3.0 mm, 4.3 mm, and 5.0 mm were inserted, respectively. Subsequently, the switched abutments for the respective platform were positioned on the implants. Then, the samples were positioned on a universal testing machine and experienced a vertical load of 190N on the abutment, with a head speed of 1 mm/min. For each specimen, three recordings were made, and the average was considered for each. To investigate how platform switching and implant diameter affect primary implant stability and bone strain around immediately loaded implants, an *in vitro* experimental test was conducted that included Periotest and strain gauge analyses. The acquired data was examined with one-way ANOVA and post hoc Tukey test.

## Results:

With vertical loading on D2, the most significant strain occurred with 3.0 mm on both the buccal and lingual sides, whereas 4.3 mm resulted in moderate strain on the lingual side. For D3 bone type under vertical loading, the maximum strain occurred with 3.0 mm on both the buccal and lingual sides (Table 1 - see PDF, 2 - see PDF). When comparing the groups, it is evident that for every diameter, the buccal strain exceeds the lingual strain significantly (p < 0.001), regardless of the diameter and bone density. The 5.0 mm implant exhibited the lowest PTV (Table 3 - see PDF), signifying the greatest primary implant stability, with no significant difference observed between the 4.3 and 3.0 implants. All three groups of implants demonstrated good primary stability, which permitted the next phase of the experiment (strain gauge analysis) to be conducted.

## Discussion:

The long-term success of osseointegrated dental implants depends on many factors. Among these, maintaining the crestal bone is still the primary principle. Creastal bone loss (CBL) occurring around the neck of dental implants is a prevalent issue post-implant placement, affecting the implant's future success [15]. Various implant designs have been proposed to counteract the impact of microleakage. Among these, platform switching (with or without a Morse taper/conical connection), utilizing both standard and reduced implant diameters, and adjusting the position of the implant-abutment junction to align with the alveolar bone crest [14]. Increased occlusal load on the prosthetic element results in crestal bone loss around the implant, leading to failure and increased stress at the implant-bone interface. It has been discovered that platform switching reduces or eliminates any predictable post-restoration bone remodeling at the crest [6]. According to the present research, implants with 3.0 mm diameter have bone strain values with platform switched are considerably higher (p < 0.01). The findings further suggest that the values of bone strain on the buccal cortical plate are higher than those on the lingual cortical plate. The hypothesis of reduced peri-implant crestal bone loss when implants were restored according to the platform switching concept was not confirmed by Enkling *et al.* from a randomized clinical trial [10]. Mukherjee *et al.* assessed the strain generated in D2 and D3 types of bones under vertical loading from platform switch implants of varying diameters. The conclusion was that the implant with a narrow diameter produces greater strain than those with diameters of 4.2 and 5.0 mm, respectively [6]. This is linked to our results. Using finite element analysis (FEA), Yadav *et al.* examined the stress distribution in platform-switched and non-platform-switched implants located in D2 (mandible) and D3 (maxilla) bones under both axial and oblique loading. Their conclusion was that platform switching enhanced stress distribution and lowered crestal bone stress in D2 and D3 bones, particularly under oblique loading [16]. The results are linked to our findings. Maeda *et al.* conducted a 3D finite element analysis for single implant-retained in the mandibular region. Wherein he investigated the biomechanical benefits of platform switching and found that it moves the stress concentration away from the implant-bone interface [17]. Rasouli-Ghahroudi *et al.* conducted a comparison of the stress distribution around tapered versus cylindrical implants and examined the influence of varying abutment diameters on crestal bone stress levels. They discovered that tapered implants increased crestal bone stress during loading, while platform switching reduced the stress transferred to the crestal bone [18]. Mitra *et al.* assessed the stress distribution in and around three different implant-abutment interfaces with platform-switched and platform-matched abutments using the finite element method (FEM). It was concluded that the internal hex connection showed the highest stress. The findings indicated that platform-switched implants applied less stress on the crestal bone compared to platform-matched implants [9]. Shalash & Abdalsamad assessed crestal bone loss (CBL) in the posterior molar area between tissue-level implants with platform matching abutments and bone-level implants utilizing conical/platform switched hybrid abutments. At the 1-year mark post-loading, both implant designs exhibited minimal CBL. Compared to tissue level implants, bone level implants with a platform switched conical hybrid connection exhibited reduced CBL [15]. Using finite element analysis, Javiya *et al.* assessed the distribution of stress surrounding implants with various designs. They arrived at the conclusion that stress distribution in the surrounding bone is significantly influenced by implant design. Stress concentration in the cancellous bone is reduced by tapered implants [19]. Manas *et al.* suggested for use of Osstell (resonance frequency analysis) and AnyCheck (damping capacity) devices. These devices showed similar performance in assessing primary and secondary implant stability [20]. Bera *et al.* concludes that cortical bone, especially high-density cortical bone, significantly contributes to primary implant stability compared to trabecular bone [21]. The limitations of this research are that it was an *in vitro* study with a limited sample size. More research is necessary to confirm the findings.

## Conclusion:

Implants with a diameter of 3.0 mm generate higher strain values on D2 and D3 bones compared to those with diameters of 4.3 mm and 5.0 mm, respectively. Hence, the buccal exhibited greater values of bone strain compared to the lingual side.
